# Acute Neurologic Decline in the ED: A Case Report on Recognizing ARIA in the Age of Anti-Amyloid Therapy

**DOI:** 10.5070/M5.61894

**Published:** 2026-10-31

**Authors:** Christopher Blanton, Joel Hines, Joshua Ginsburg, Samuel Parnell

**Affiliations:** * UT Southwestern, Department of Emergency Medicine, Dallas, TX

## Abstract

Anti-amyloid beta (Aβ) immunotherapies are an emerging treatment for Alzheimer’s disease (AD) with a rare but significant risk of amyloid-related imaging abnormalities (ARIA). This visual case report describes a patient with lecanemab-induced ARIA to familiarize emergency providers with this critical adverse effect of novel anti-amyloid therapies.

An 85-year-old female receiving lecanemab infusions presented to the emergency department (ED) with abruptly worsening confusion, an unsteady gait, and new left-sided deficits, including homonymous hemianopia and hemineglect. A non-contrast head computed tomography (CT) revealed extensive right parieto-occipital vasogenic edema, prompting magnetic resonance imaging (MRI), which confirmed extensive ARIA with edema (ARIA-E) on fluid-attenuated inversion recovery (FLAIR) sequences and microhemorrhages (ARIA-H) on T2-weighted sequences. Lecanemab was discontinued, and the patient experienced partial neurologic recovery after a four-day admission. However, she was discharged with persistent functional limitations, including an inability to drive and a need for caregiver assistance.

This case underscores the need for emergency physicians to maintain a high index of suspicion for ARIA in patients on anti-Aβ immunotherapies who present with new neurologic symptoms. Definitive diagnosis requires MRI, and management warrants prompt neurologic consultation.

**Topics:**

Alzheimer’s, dementia, neurology, medication adverse effects, ARIA.

**Figure:**
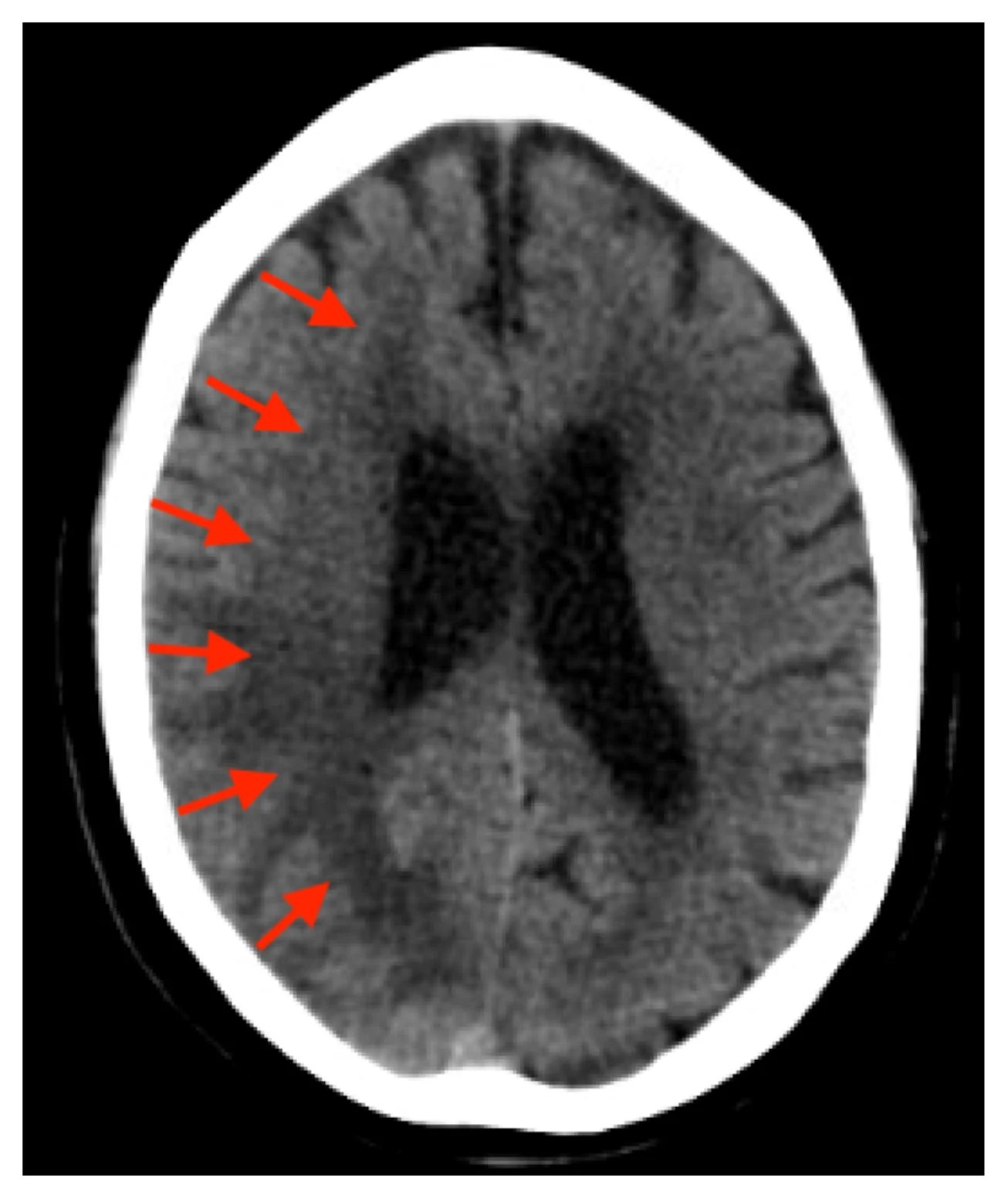


**Figure:**
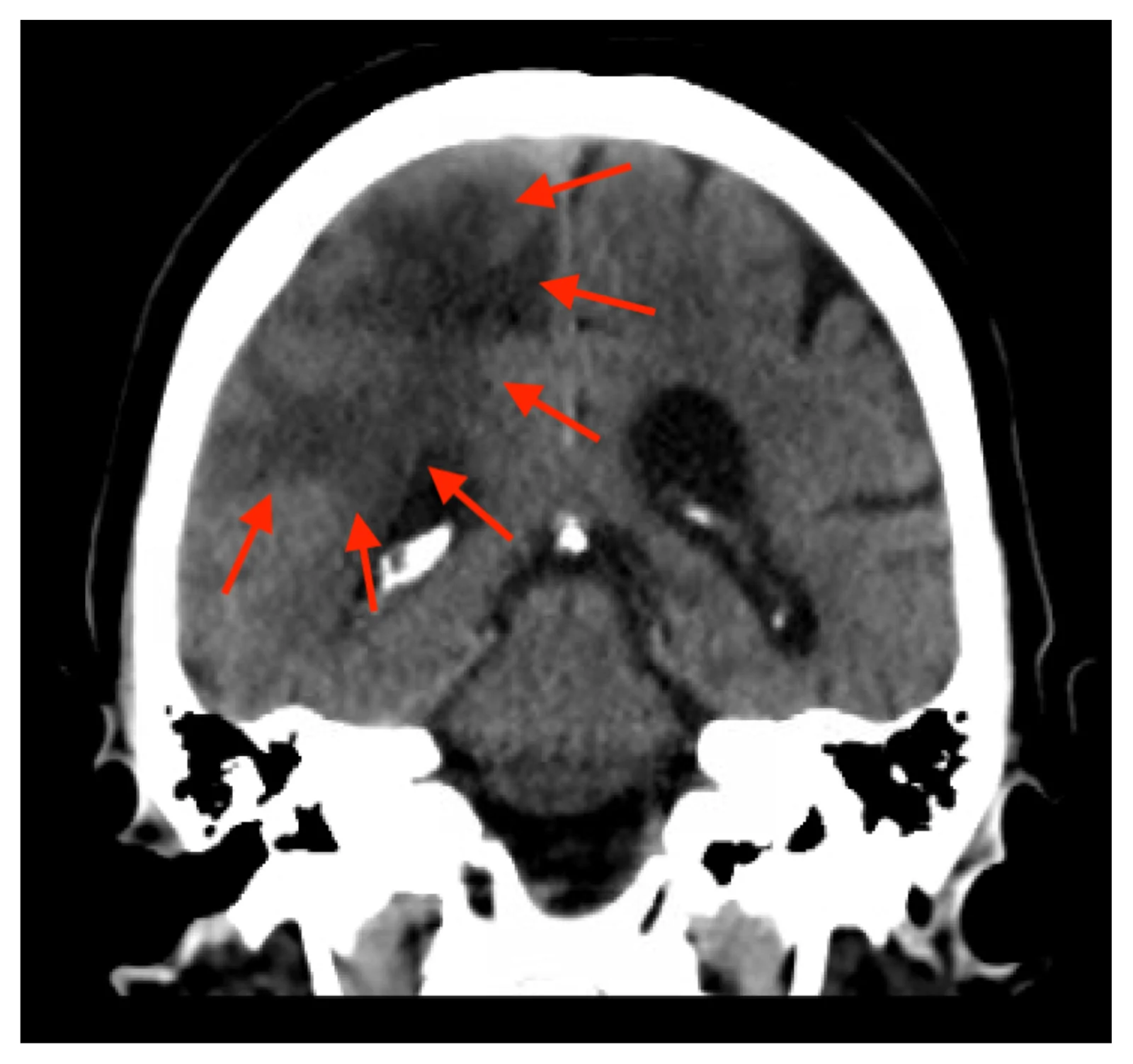


**Figure:**
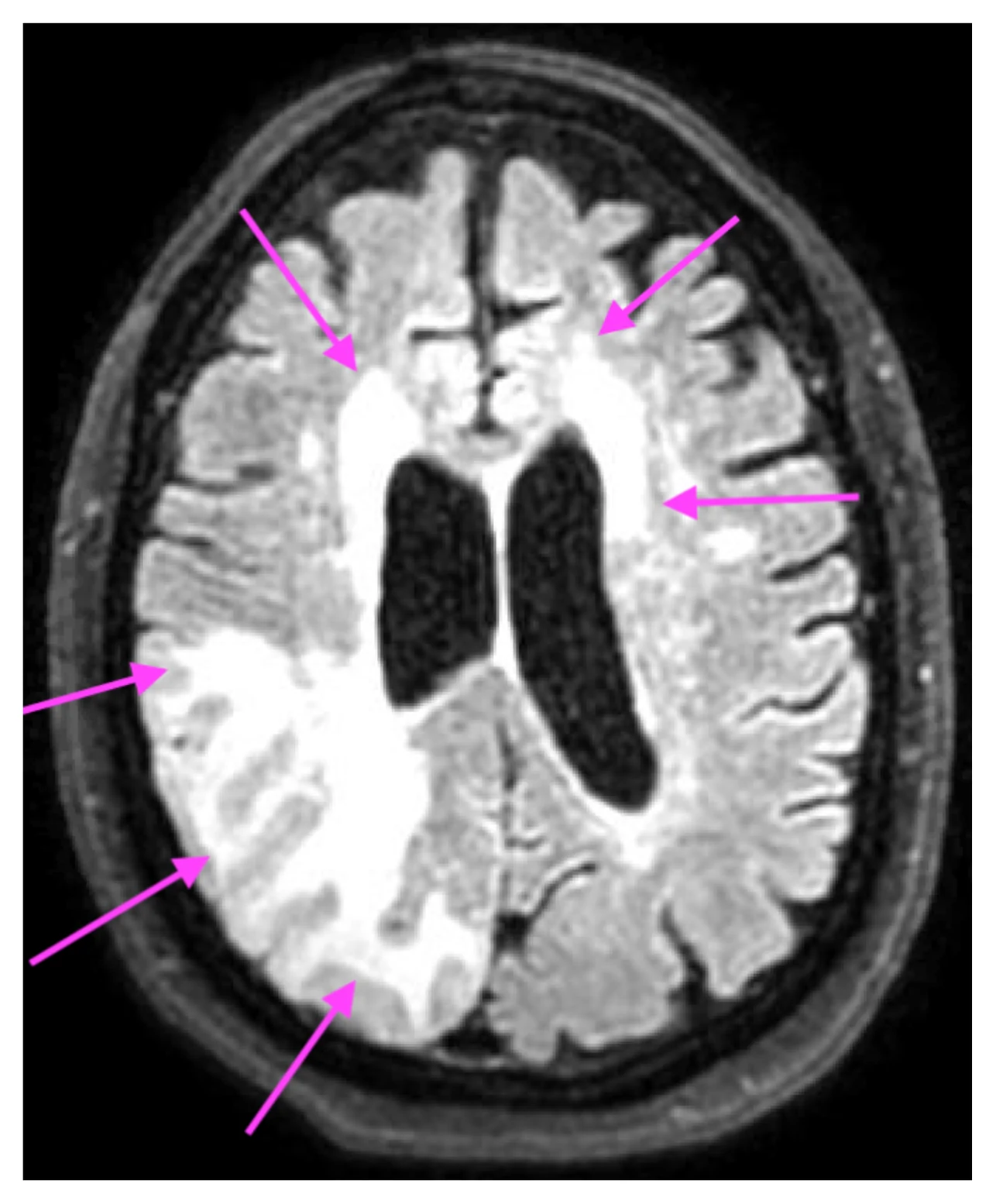


**Figure:**
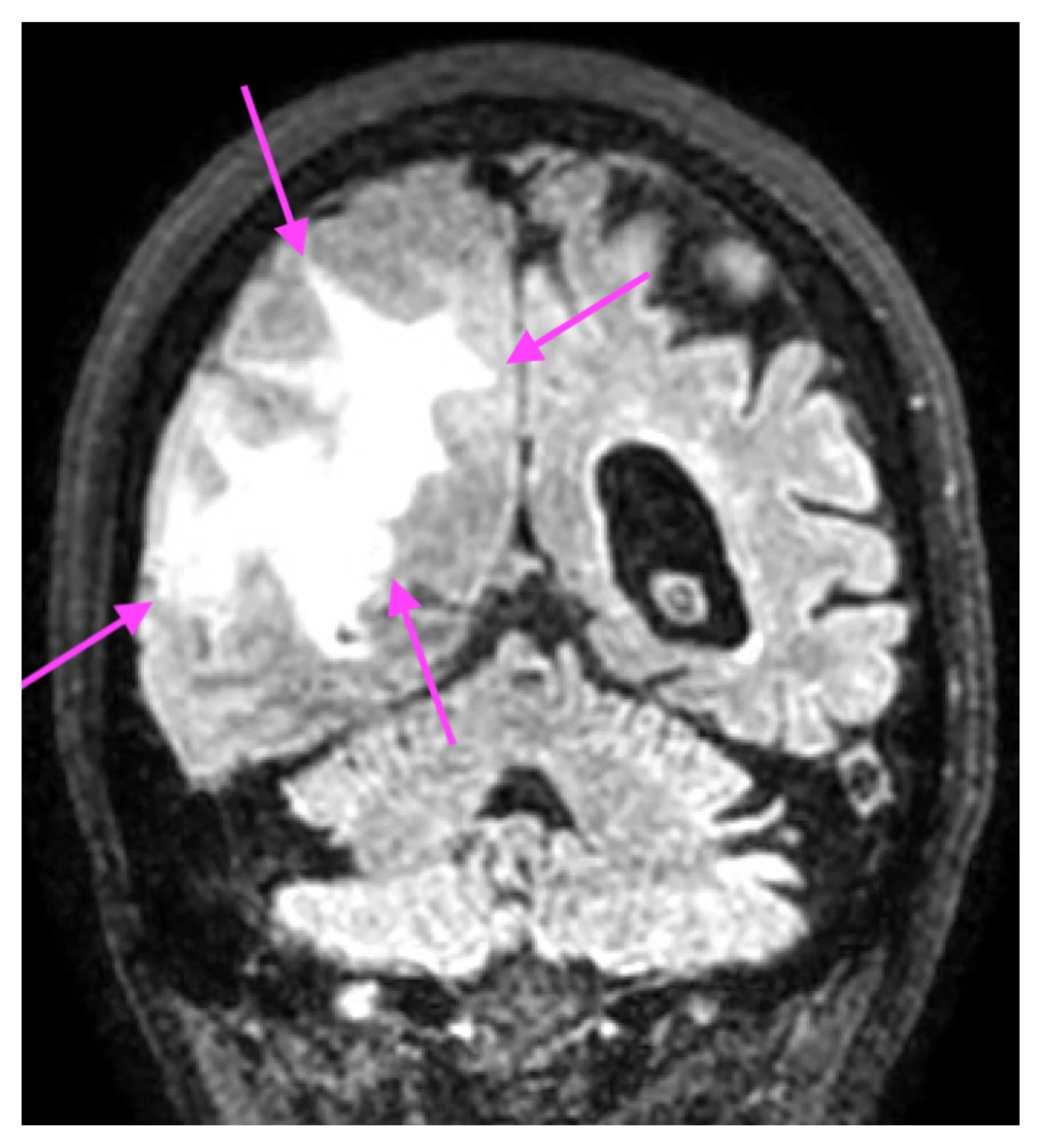


**Figure:**
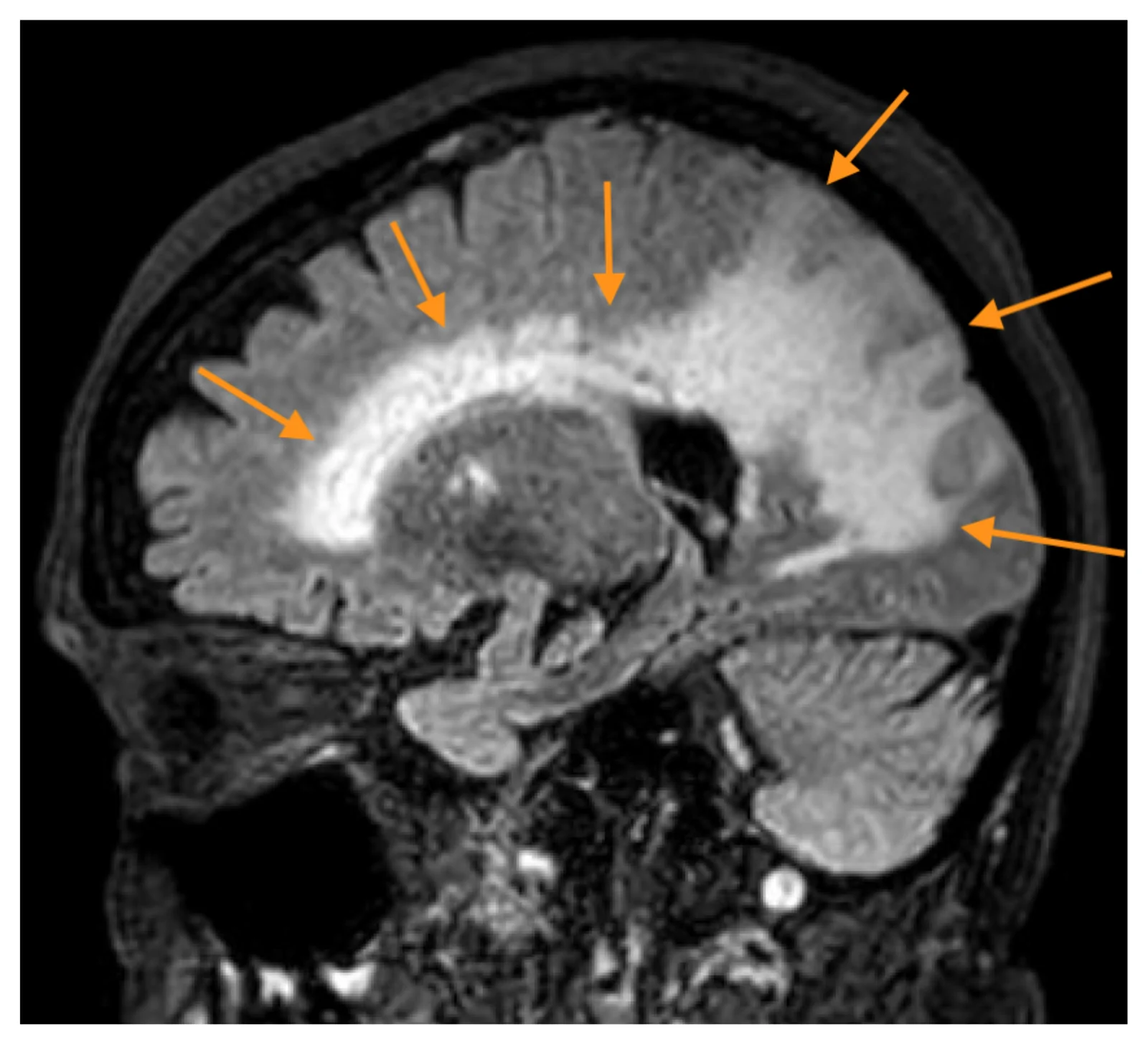


**Figure:**
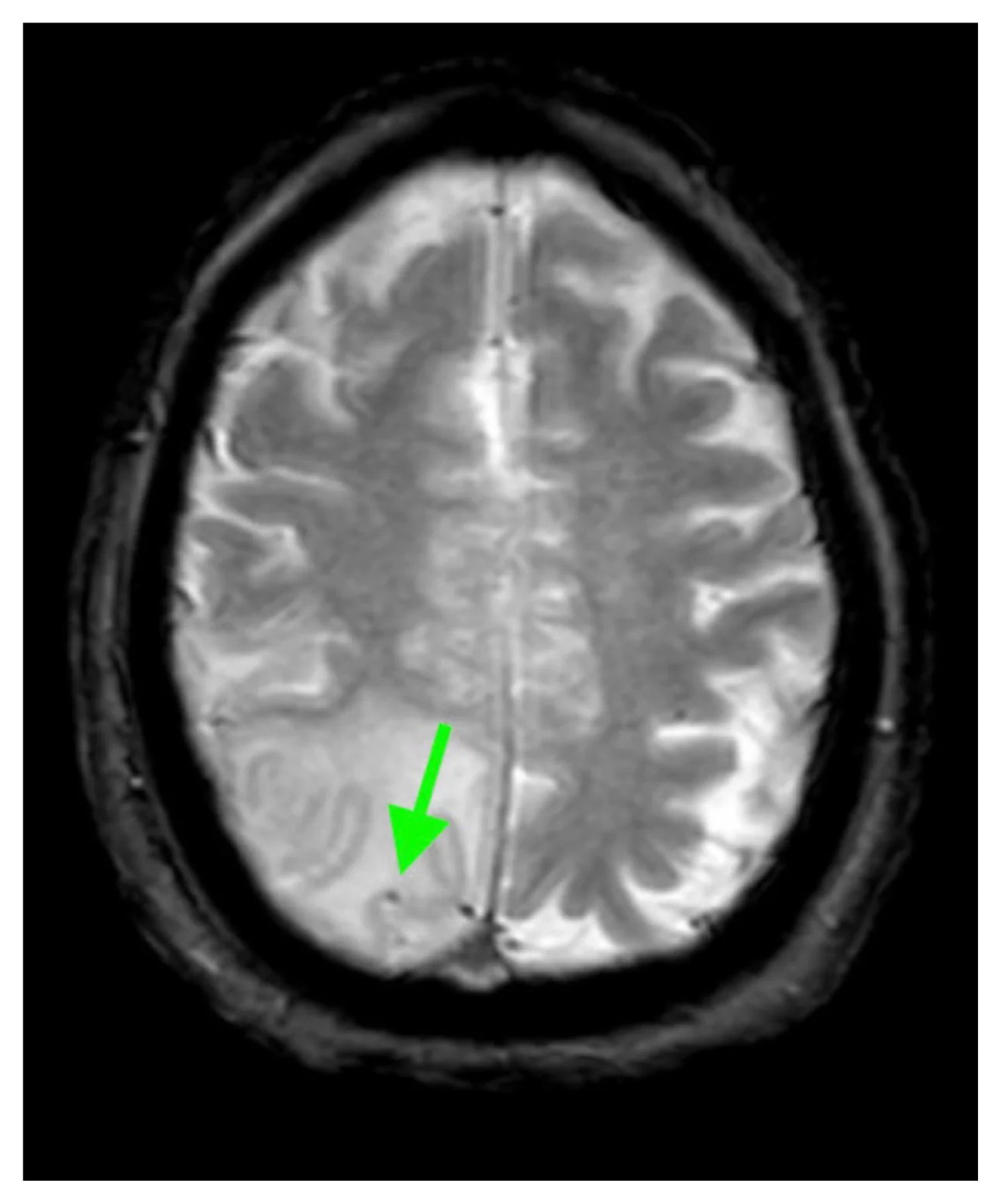


**Figure:**
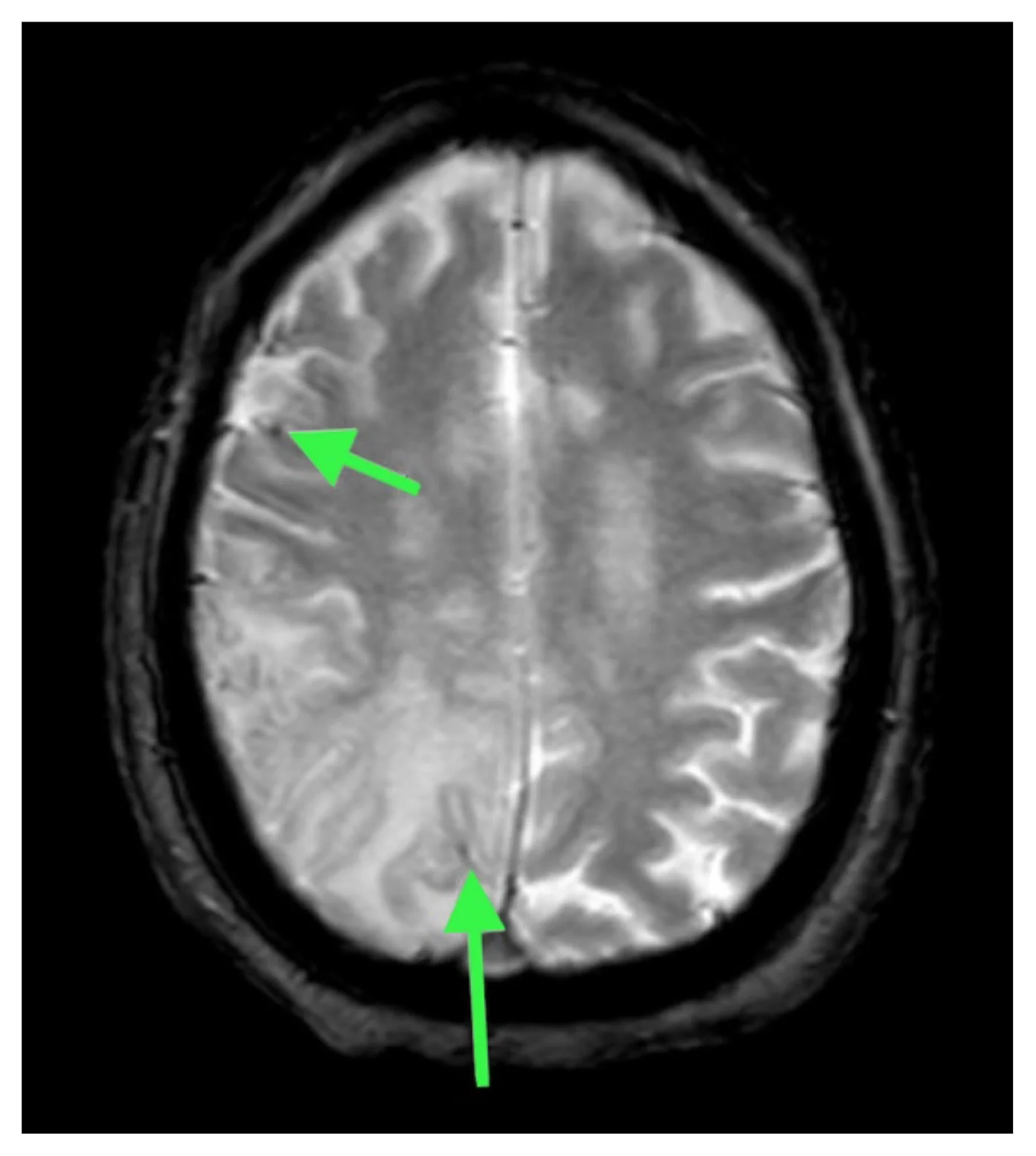


## Brief introduction

Anti-Aβ immunotherapies are an innovative class of treatments for AD. One leading hypothesis of AD proposes that extracellular β-amyloid accumulation contributes to downstream tau pathology, neurodegeneration, and progressive cognitive decline. Anti-amyloid monoclonal antibodies bind aggregated β-amyloid and facilitate its clearance through microglial-mediated immune mechanisms.^1^ While these therapies have shown promise in slowing the burden of AD, they also carry risks of serious adverse events. The most clinically significant and class-defining adverse event is ARIA, which is classified into two main types: ARIA-E, characterized by edema, and ARIA-H, characterized by hemosiderin deposits with or without microhemorrhage.^1^ While most patients with ARIA are asymptomatic, some can present with headache, confusion, dizziness, neurologic deficits, and seizures.^2^ Given its novelty, there is little discussion in the emergency medicine literature on ARIA. This case report describes a patient seen in our ED diagnosed with both ARIA-E and ARIA-H. This report also aims to provide a brief overview of ARIA for emergency medicine providers.

## Presenting concerns and clinical findings

An 85-year-old female with a pertinent history of hypertension, hyperlipidemia, and dementia secondary to AD presented to the ED with a family member after several days of increasing confusion and weakness, which abruptly worsened that morning. She had a past medical history of mild AD and was on biweekly lecanemab infusions. At the time of evaluation, she had received eight lecanemab infusions over the preceding six months. According to her family, she experienced mild confusion and subtle changes in her daily routine for approximately three days prior to arrival. On the morning of presentation, her symptoms acutely progressed to include left-hand incoordination, an unsteady gait with leftward leaning, and possible left visual field neglect.

The physical exam while in the ED was remarkable for orientation only to person and place. The neurologic exam revealed word-finding difficulties, left homonymous hemianopia, left hemineglect, left-sided facial numbness, and mild dysmetria in the left hand. Initial labs were unremarkable, except for urinalysis revealing a urinary tract infection, which was appropriately treated.

## Significant findings

Computed tomography of the brain without contrast revealed extensive right parieto-occipital vasogenic edema (red arrows on the CT brain axial and coronal images). The finding of vasogenic edema led to further investigation with MRI of the brain without contrast. This revealed extensive hyperintense areas in the right parietal and occipital lobes on T2-weighted (purple arrows on the T2-weighted MRI axial and coronal images) and FLAIR sequences (orange arrows on T2 FLAIR sagittal image). These hyperintensities represent vasogenic edema, consistent with ARIA-E.^3^ MRI also revealed five focal low-intensity lesions in the right cerebral hemisphere on T2-weighted-GRE (gradient echo sequence) (green arrows on the T2-GRE axial images). These low-intensity foci represent microhemorrhages, consistent with ARIA-H.^3^

## Patient course

This patient was admitted to the general neurology service with new neurologic deficits and MRI findings concerning for ARIA-H and ARIA-E, attributed to the use of lecanemab. Upon admission, the lecanemab was discontinued. Over the subsequent days, the patient’s neurologic symptoms began to improve. Given the neurologic improvement, the neurology team decided not to administer steroids.

The patient was admitted for a total of four hospital days and required physical, occupational, and speech therapy before discharge with home health. At her clinic follow-up, she exhibited meaningful neurologic recovery. However, she continued to experience occasional flashes in the left visual field and intermittent imbalance. Cognitively, the patient reported significant improvement but continued to feel slightly more disorganized compared to before the event. Although the patient regained the ability to perform some activities of daily living after diagnosis and treatment of ARIA-H and ARIA-E, functional deficits persisted, precluding driving and necessitating post-discharge caregiver assistance.

## Discussion

Alzheimer’s disease is the most common type of dementia. In the United States alone, there are currently over six million people with AD. This number is projected to reach nearly 14 million people by 2060, with an estimated future healthcare cost of over 1 trillion dollars by 2050.^4^ Considering the aging populations in developed countries and growing global burden of AD, it is reasonable to assume there will be increasing demand for and use of anti-Aβ immunotherapies. To date, the U.S. Food and Drug Administration (FDA) has approved three anti-Aβ monoclonal antibodies: aducanumab, lecanemab, and donanemab. These monoclonal anti-amyloid antibodies have been shown to reduce β-amyloid deposition through microglial activation and have been associated with cognitive benefits in AD clinical trials. This is important because one current hypothesis of AD development is that abnormal accumulation of β-amyloid protein may be a key event triggering tau pathology and neurodegeneration. However, the transient inflammatory response from anti-Aβ monoclonal antibodies accelerating clearance of β-amyloid is also thought to increase blood-brain barrier permeability, leading to vasogenic edema (ARIA-E) and, in some patients, microhemorrhage (ARIA-H). This proposed mechanism explains the close relationship between ARIA and underlying cerebral amyloid angiopathy. 5–7 While these therapies have shown promise in slowing the burden of disease in AD, they also carry risks of serious adverse events.

The adverse effect profiles of these anti-Aβ immunotherapy agents are broadly similar, with ARIA representing the most clinically significant and class-defining toxicity. Although all FDA-approved anti-amyloid monoclonal antibodies carry a risk of both ARIA-E and ARIA-H, lecanemab appears to be associated with a lower risk of severe ARIA and intracerebral hemorrhage than aducanumab and donanemab, while demonstrating a similar or slightly higher incidence of infusion-related reactions and other mild-to-moderate adverse events.^5^–^7^ Non-ARIA adverse effects are also common and vary by agent. Compared with donanemab, lecanemab is associated with a higher rate of infusion-related reactions (26.4% vs. 8.7%), as well as headache (11.1%), falls (10.4%), and dizziness (5.5%).^8^

The strongest risk factor for developing ARIA is an apolipoprotein E (APOE) E4 genotype, with homozygotes having a markedly increased risk.^9^ Because APOE E4 carriers have greater parenchymal and vascular β-amyloid deposition, they may undergo more extensive amyloid clearance during anti-amyloid monoclonal antibody therapy. This accelerated clearance is thought to transiently increase vascular permeability and exacerbate underlying cerebral amyloid angiopathy (CAA), predisposing patients to proteinaceous fluid leakage (ARIA-E) and microhemorrhage (ARIA-H).^9^ Interestingly, the patient in this case did have the APOE E4 genotype, increasing her risk of ARIA. Other risk factors include baseline cerebral microhemorrhages and superficial siderosis, reflecting underlying cerebral amyloid angiopathy (CAA), a higher treatment dose of anti-amyloid antibody, elevated mean arterial pressure, and greater amyloid plaque burden.^9^–^11^ Age, female sex, and comorbid cerebrovascular disease may contribute, but their independent effects are less robust after multivariable adjustment.^10^,^11^

The pathophysiology of ARIA is believed to be linked to underlying CAA, in which Aβ deposits accumulate in the cerebral vasculature, compromising the blood-brain barrier (BBB) and increasing the risk of vascular permeability and cerebral edema.^12^,^13^ Aβ monoclonal antibodies trigger an immune response against Aβ deposits, causing microglial activation with subsequent inflammation, further compromising the BBB.^14^ The dose-dependent incidence of ARIA may suggest that a greater degree of Aβ removal leads to greater vasculature compromise.^1^ Furthermore, 49% of ARIA-E cases can have an associated appearance of ARIA-H, suggesting a common pathophysiologic mechanism. 15 ARIA most commonly develops early during treatment, often during the first several infusions, although delayed presentations have also been reported.^16^ In addition, this phenomenon appears to occur with some frequency in patients on anti-Aβ immunotherapy. Clinical trials of lecanemab have reported ARIA-E in approximately 12–13% of treated patients, whereas symptomatic ARIA is considerably less common.^6^

While non-specific findings such as vasogenic edema or microhemorrhages may be seen on CT imaging, MRI is required for the diagnosis of ARIA.^12^ Most cases tend to be asymptomatic, and symptoms—when present—tend to be nonspecific and overlapping with other neurologic conditions. The most common symptoms of ARIA include headache, confusion, dizziness, visual disturbances, and nausea.^2^ More rare symptoms include seizures, encephalopathy, or focal neurological deficits. In exceptional cases, ARIA can result in hospitalization, permanent disability, or death.^11^, ^16^–^20^ ARIA-E is more likely to be symptomatic, with symptoms such as headache, confusion, and altered mental status, whereas ARIA-H can occasionally present with focal neurological symptoms or, rarely, acute hemorrhagic events.11,16–20

Diagnosis requires MRI with the appropriate sequences, as recommended by the American College of Radiology and the American Academy of Neurology.^3^,^19^ ARIA-E will show vasogenic edema and/or sulcal effusions on MRI, specifically as hyperintensity on T2-weighted or FLAIR sequences. ARIA-H will show cerebral microhemorrhages, macrohemorrhages, or superficial siderosis detected on T2-weighted gradient echo or susceptibility-weighted imaging (SWI) MRI sequences.^3^

The differential diagnosis for patients presenting with acute focal neurologic deficits and altered mental status is broad and includes acute ischemic stroke, intracranial hemorrhage, seizure with a postictal state, infection, metabolic derangements, and medication-related adverse effects. This case also highlights the potential for diagnostic anchoring. Although the patient had a urinary tract infection that could reasonably explain worsening confusion in an older adult, her focal neurologic deficits and neuroimaging findings prompted further evaluation, ultimately leading to the diagnosis of ARIA. Similar to previously reported symptomatic cases, our patient presented with nonspecific neurologic symptoms, including confusion, visual disturbances, and focal neurologic deficits, underscoring the diagnostic challenge of ARIA in the emergency department.^8^,^19^ Emergency clinicians should therefore maintain a high index of suspicion for ARIA in patients receiving anti-amyloid therapy who develop new neurologic symptoms, even when an alternative diagnosis appears plausible.

Patients on anti-Aβ immunotherapy presenting to the ED with any new neurologic symptoms should receive rapid neurology consultation and MRI evaluation. In patients with minor symptoms like headache, dizziness, or nausea, the decision to obtain an MRI may be deferred to neurology consultants. Although most cases resolve simply with drug cessation, severe or symptomatic ARIA may benefit from high-dose corticosteroids.^3^ Other supportive measures in the ED may include treating seizures, infusion reactions, or anaphylaxis from anti-Aβ immunotherapy infusions through standard protocols.^19^ Additionally, emergency clinicians should also be mindful of the increased risk of hemorrhagic conversion with thrombolytics and the need to exercise caution with anticoagulation in these patients.^19^ If a patient is started on anticoagulation for deep vein thrombosis or pulmonary embolism, anti-amyloid medications should be held and discussed with the prescribing provider if at all possible. Furthermore, patients on anti-Aβ immunotherapy should ideally receive an MRI shortly after initiating anticoagulation. Patients on anti-Aβ immunotherapy generally require regular surveillance, emphasizing the importance of ensuring close outpatient follow-up with neurology.

In conclusion, given the novelty of anti-Aβ immunotherapies and the fact that ARIA can present with nonspecific symptoms, diagnosis may be easily missed in the ED. This case highlights the need for ED providers to be aware of anti-Aβ immunotherapies and their adverse effects.

## Supplementary Information




























